# Utility of the *SERPINC1* Gene Test in Ischemic Stroke Patients With Antithrombin Deficiency

**DOI:** 10.3389/fneur.2022.841934

**Published:** 2022-06-03

**Authors:** Seondeuk Kim, Woo-Jin Lee, Jangsup Moon, Keun-Hwa Jung

**Affiliations:** ^1^Department of Neurology, Seoul National University Hospital, Seoul, South Korea; ^2^Department of Genomic Medicine, Seoul National University Hospital, Seoul, South Korea; ^3^Program in Neuroscience, Seoul National University College of Medicine, Seoul, South Korea

**Keywords:** genetic strokes, *SERPINC1* mutation, ischemic stroke, pathogenic variant, antithrombin (AT) deficiency

## Abstract

**Objective:**

Antithrombin (AT) plays a critical role in the coagulation system, and its deficiency induces hypercoagulability. AT deficiency is caused not only by inherited variants in the *SERPINC1* gene but also by acquired conditions. Therefore, AT deficiency alone could not ensure the presence of the *SERPINC1* mutation. We evaluated the utility of the *SERPINC1* gene test in ischemic stroke, an important clinical type of arterial thrombosis.

**Methods:**

This retrospective, observational study investigated symptomatic patients who underwent the *SERPINC1* gene test because of decreased AT activity (<80%) during 2009-2021 at a tertiary hospital. For the detection of sequence variants in the *SERPINC1* gene, direct Sanger sequencing and multiplex ligation-dependent probe amplification were performed. The phenotypes of patients with SERPINC1 gene mutations were examined, and the conditions associated with the pathogenic variants were analyzed.

**Results:**

In our cohort (*n* = 19), 13 of 19 patients (68.4%) had the pathogenic variant of the *SERPINC1* gene. Ischemic stroke (*n* = 7) was significantly associated with the pathogenic variants (*p* = 0.044), and the pathogenicity detection rate was 100%. For any kind of arterial thrombosis (*n* = 8), the detection rate of the pathogenic variant was 87.5%, but was not statistically significant (*p* = 0.177). The detection rates of the pathogenic variant in ischemic stroke or arterial thrombosis groups were both higher than those in the venous thrombosis-only group (54.5%).

**Conclusion:**

The *SERPINC1* gene test was useful in determining the cause of AT deficiency-related arterial thrombosis, especially ischemic stroke. We propose the diagnostic flow of *SERPINC1*-related ischemic stroke.

## Introduction

Antithrombin (AT) is an inhibitor of a serine protease that plays a critical role in inactivating coagulation factors such as thrombin and activated factors X, IX, XI and XII (1). A small change in AT level or activity can result in a significant alteration in coagulation function. Although AT deficiency is caused by acquired conditions such as sepsis, liver cirrhosis, nephropathy, and disseminated intravascular coagulation (2), several mutations of the AT encoding gene *SERPINC1*, which is located on chromosome 1q23-25 and consists of seven exons, have also been identified to be associated with AT deficiency and various coagulopathies (3). Therefore, AT deficiency alone could not reflect *SERPINC1* mutation.

AT deficiency is known to be associated with venous thromboembolism. However, the association between AT deficiency and arterial thromboembolism was not clearly appreciated, possibly due to its relatively lower incidence, albeit the tendency to develop any kind of thrombosis (4–6). The current evidence could not support clinicians in testing the AT and *SERPINC1* genes in arterial thrombosis, contrary to venous thrombosis. In this regard, the utility of the *SERPINC1* gene test in ischemic stroke remains elusive, even though hypercoagulability is one of the risk factors for arterial thrombosis. In this study, we hypothesized that arterial thrombosis associated with altered AT would have comparable detectability of pathogenic *SERPINC1* variants with venous thrombosis. Our study is the first report about the utility of the *SERPINC1* gene test in ischemic stroke, an important clinical type of arterial thrombosis.

## Method

### Patient Inclusion and Acquisition of Clinical Data

This retrospective, observational study investigated patients who underwent the *SERPINC1* gene test on suspicion of inherited AT deficiency during 2009-2021 at a tertiary hospital. The indications for testing AT activity included venous thrombosis events, such as deep venous thrombosis (DVT), pulmonary thromboembolism (PTE), cavernous venous thrombosis (CVT), and splanchnic vein thrombosis (SVT), without an evident provoking factor (e.g., prolonged immobilization, cancer, pregnancy, inflammatory bowel disease). Moreover, ischemic stroke with atypical etiology, including young age stroke (<45 years old), recurrent stroke refractory to treatments, and embolic stroke without a definite source, was included as an indication for this test. When AT deficiency was detected, a *SERPINC1* gene test was performed. The 23 symptomatic patients with decreased AT activity were identified using the electrical medical record system. Among them, four patients didn't undergo the *SERINC1* gene test. From a total of 19 patients, demographics, clinical manifestations, underlying disease, family history, laboratory and radiological findings, and genetic information were collected. Other coagulopathy statuses, including protein C, protein S, anticardiolipin antibody, lupus anticoagulant and homocysteine, were also examined.

### AT Analysis

Using a HemosIL Liquid Antithrombin AT III kit and ACL TOP 750 CTS (Werfen®), AT activities were measured automatically. The citrated plasma of the patient was incubated with Factor Xa reagent and an excess of heparin. The residual FXa activity was quantified using the para-nitroaniline released from the reaction between the FXa and synthetic chromogenic substrate. Then, AT activity was inversely proportional to the residual FXa activity (7, 8). In healthy individuals, AT activities range from 83 to 128%. Abnormal AT activity was defined as <80%.

### Genetic Tests

DNA samples were extracted from the patients' blood samples. Mutations in the *SERPINC1* gene were analyzed by two methods. For the detection of sequence variants, polymerase chain reaction (PCR) and direct Sanger sequencing of *SERPINC1* exons 1-7 were conducted. Deletion or duplication was detected by multiplex ligation-dependent probe amplification (MLPA) of the *SERPINC1* gene. The sequences were aligned to the reference sequence (NC_000001.9, NM_000488.2).

### Pathogenicity of *SERPINC1* Variant

Clinical significance (i.e., pathogenicity) of the *SERPINC1* variant was decided according to the American College of Medical Genetics and Genomics (ACMG) 2015. The human gene mutation database (HGMD) was applied as a mutation database (http://www.hgmd.cf.ac.uk/ac/gene.php?gene=SERPINC1). The *in silico* programs used were SIFT, Polyphen2, and Mutation Taster. The Korean reference genome database (http://152.99.75.168/KRGDB) was used as a population database.

### Statistical Analyses

Patients were stratified based on the clinical reason for the test and the laboratory findings. Fisher's exact test was applied to identify the conditions associated with the pathogenic variant of *SERPINC1*. Using SPSS version 27, all analyses were performed. A *P* value <0.05 was considered statistically significant.

### Ethics Statement

The Institutional Review Board (IRB) of Seoul National University approved the antithrombin deficiency and ischemic stroke cohort study (H-2105-031-1216). This study was conducted from a retrospective chart review. For this study, the IRB granted the absence of informed consent. We deidentified the information from medical records for the privacy of subjects.

## Results

### Study Population

Of 23 patients with thrombotic events related to altered AT activity, a total of 19 patients underwent targeted sequencing and MLPA of the *SERPINC1* gene. Thromboembolic events included both cerebral thromboembolism (*n* = 10) and non-cerebral thromboembolism (*n* = 9). The mean age of the patients was 34.8 years (range 4 to 77), and 11 patients [57.9%] were male (Table 1). Mutations in the *SERPINC1* gene were found in 13 of 19 patients (68.4%). The patients were categorized into three groups according to the clinical situation for the test: arterial thrombosis (*n* = 6); venous thrombosis (*n* = 11); and mixed thrombosis (*n* = 2). Analysis was performed initially for two groups: arterial thrombosis (*n* = 8) and venous thrombosis (*n* = 13) and further for more specific groups: ischemic stroke (*n* = 7) and venous thrombosis only (*n* = 11).

**Table 1:** Analysis of antithrombin III deficiency patients with *SERPINC1* gene test and pathogenic variant detection rate.

	***SERPINC1* gene test**	**Pathogenic variant**	**Detection rate**	***p*-value**
**Patients**	*n =* 19	*n =* 13		
Study of Age (yrs)	34.8 (sd 26.7)	33.5 (sd 23.1)		
Sex (Male)	11 (57.9%)	8 (61.5%)	72.7%	>0.999
Sex (Female)	8 (42.1%)	5 (38.5%)	62.5%	>0.999
Family history of hypercoagulability	2 (10.5%)	2 (15.4%)	100%	>0.999
**Cause of test**				
Venous thrombosis and/or arterial thrombosis	13 (68.4%)	8 (61.5%)	61.5%	0.605
Venous thrombosis only	11 (57.9%)	6 (46.2%)	54.5%	0.177
Arterial thrombosis	8 (42.1%)	7 (53.8%)	87.5%	0.177
Ischemic stroke	7 (36.8%)	7 (53.8%)	100%	0.044*
**Accompanying** **disease or abnormality**				
Heart disease	2 (10.5%)	2 (15.4%)	100%	>0.999
IVC abnormality	2 (10.5%)	2 (15.4%)	100%	>0.999
Kidney disease	2 (10.5%)	2 (15.4%)	100%	>0.999
HTN	3 (15.8%)	2 (15.4%)	66.7%	>0.999
Positive APS antibody	5 (26.3%)	4 (30.8%)	80%	>0.999

yrs, years; sd, standard deviation; IVC, inferior vena cava; HTN, hypertension; APS, antiphospholipid syndrome. *p-value <0.05.

### Conditions Associated With Pathogenic Variants of the *SERPINC1* Gene

To identify the conditions related to the pathogenic variants, we analyzed the association and detection rate of pathogenic variants in groups with different clinical situations (Table 1). The analyses for venous thrombosis-only group or combined with arterial thrombosis showed no significant association with the pathogenic variants (ratio of the patients with the variant and the specific condition to all patients with the variant vs. the patients with the gene test and specific condition to all patients with the gene test; 8/13 [61.5%] vs. 13/19 [68.4%], *p* = 0.605; 6/13 [46.2%] vs. 11/19 [57.9%], *p* = 0.177, respectively), and the detection rate of the venous thrombosis-only group (54.5%) was relatively lower than that of the arterial thrombosis groups. For arterial thrombosis, including superior mesenteric occlusion, the detection rate was 87.5%, but the difference was not statistically significant (7/13 [53.8%] vs. 8/19 [42.1%], *p* = 0.177). Ischemic stroke was significantly associated with pathogenic variants (7/13 [53.8%] vs. 7/19 [36.8%], *p* = 0.044), and the pathogenicity detection rate was 100%.

### The Distribution and Phenotypes of the *SERPINC1* Variants

In our series (Supplementary Table S1), the most common variant was *c*. *442T*>*C, p. Ser148Pro* (4/13, 30.8%), and the variant manifested only with ischemic stroke (4/4 100%). *c. 235C*>*T, p. Arg79Cys* was the second most common variant (3/13, 23.1%). The phenotypes of the *c.235C*>*T, p*. The *Arg79Cys* variant included DVT (1/3, 33.3%) and ischemic stroke (3/3, 100%). This mutation showed much lower AT activity (19.00, range 7 to 27) than other mutations. It might result from two patients with homozygosis.

### Phenotypes of the *SERPINC1* Variant Related Ischemic Stroke

The patients with *SERPINC1* variant-related ischemic stroke were divided into two groups with embolic infarction patterns (*n* = 4) or episodes associated with large artery disease (*n* = 3) (Table 2). In the embolic infarction group, two patients were young stroke patients, consisting of one whose embolism was of unknown origin and the other who had atrial septal defect with DVT, and the remaining two older patients were one with unknown embolic origin and the other with atrial fibrillation (AF). In the patient with AF, subacute infarction with hemorrhagic transformation, CVT, vasculitis were included as differential diagnoses. After extensive workup, including digital subtraction angiography, brain biopsy, laboratory tests associated with vasculitis, the final diagnosis of cerebral infarction was made, and AT deficiency was confirmed. In the large artery disease group, two patients met the criteria of young age stroke. Notably, a 4-year-old girl who presented with right-hand weakness was suspected of having moyamoya syndrome involving the left distal ICA, although *RNF213* gene analysis was not performed. After several months, she experienced myocardial infarction (MI). The other 76-year-old male patient, who had vertebral artery stenosis, underwent recurrent ischemic stroke with an interval of 2–3 weeks notwithstanding antiplatelet therapy.

**Table 2:** Detailed Profile of the Ischemic Stroke patients with *SERINC1* variants.

**Patient ID**	**Arterial onset age**	**Venous onset age**	**Sex**	**AT activity**	***SERPINC1* gene**	**Certainty of pathogenicity**	**Arterial thrombosis**	**Venous event**	**Arterial ischemic symptoms**	**NIHSS**	**Brain MRI finding**	**APL**	**Protein C**	**Protein S**	**Heart Dz**	**HTN**	**DM**	**HL**
Embolic infarction																		
#1	24	24	F	27%	c.235C>T, p.Arg79Cys, homozygote	Pathogenic	Ischemic stroke	DVT	Sensory aphasia	2	Acute infarction Lt MCA inferior division Chronic infarction Lt MCA superior division Rt MCA/PCA borderzone	LA ACA Anti-β2 GP1 Ab	97	80	ASD SSS 2:1 AVB	None	None	None
#2	5	NA	M	7%	c.442T>C, p.Ser148Pro c.235C>T, p.Arg79Cys	Likely pathogenic and Pathogenic	Ischemic stroke	None (r/o KILT syndrome)	Left side weakness	10	Acute infarction Rt MCA inferior division dominant multiple territory Chronic infarction Lt MCA superior division	LA	70	91	None	None	None	None
#3	67	NA	M	43%	c.442T>C, p.Ser148Pro, heterozygote	Likely pathogenic	Ischemic stroke	None	Sensory aphasia	4	Subacute infarction Lt MCA inferior division with hemorrhagic transformation	LA	115	75	AF	None	None	None
#4	61	61	F	60%	c.1154-14G>A, (IVS5), heterozygote	Likely pathogenic	Ischemic stroke	Splanchnic vein thrombosis	Tonic-clonic seizure	1	Acute infarction Bilateral ACA	None	114	41	None	None	None	None
Large artery disease																		
#5	76	NA	M	37%	c.442T>C, p.Ser148Pro, heterozygote	Likely pathogenic	Ischemic stroke (Recurrent)	None	Sensory change >VFD	2	Acute infarction right thalamus → Both PCA involving both temporal/occipital lobe Near occlusion of both proximal PCA with poor distal visualization of distal flow	None	105	79	None	None	None	Presence
#6	44	NA	F	40%	c.442T>C, p.Ser148Pro, heterozygote	Likely pathogenic	Transient ischemic attack (Recurrent)	None	VFD Sensory change, impaired word memory	0	Lt P2 tight stenosis	None	109	88	None	None	None	Presence
#7	4	NA	F	23%	c.235C>T, p.Arg79Cys, homozygote	Pathogenic	Ischemic stroke (recurrent) → MI	None (r/o KILT syndrome)	Rt hand weakness → Lt hand weakness	1	Rt side ischemic event Chronic infarction Lt ACA territory infarction Lt distal ICA tight stenosis with collateral vessel Lt A2 stenosis Lt P2 stenosis (Moyamoya like) Lt side ischemic event Acute infarction Rt MCA superior division Rt MCA/PCA borderzone Rt distal ICA stenosis	None	82	106	None → Multi-focal MI, LVOT septal hyper-trophy	Reno-vascular HTN	None	None

*ID, identification; AT, antithrombin; NIHSS, national institute of health stroke scale; MRI, magnetic resonance imaging; APL, antiphospholipid; Dz, disease; HTN, hypertension; DM, diabetes mellitus; HL, hyperlipidemia; NA, not applicable; M, male; F, female; DVT, deep vein thrombosis; Rt., right; Lt., left; MCA, middle cerebral artery; PCA, posterior cerebral artery; ACA, anterior cerebral artery; ICA, internal carotid artery; LA, lupus anticoagulant; ACA, anti-cardiolipin antibody; GPI Ab, glycoprotein I antibody; ASD, atrial septal defect; SSS, sick sinus syndrome; AVB, atrioventricular block; KILT, kidney and inferior vena cava abnormality with leg thrombosis; AF, atrial fibrillation; VFD, visual field defect; MI, myocardial infarction; LVOT, left ventricular outflow tract*.

## Discussion

**Figure 1:**
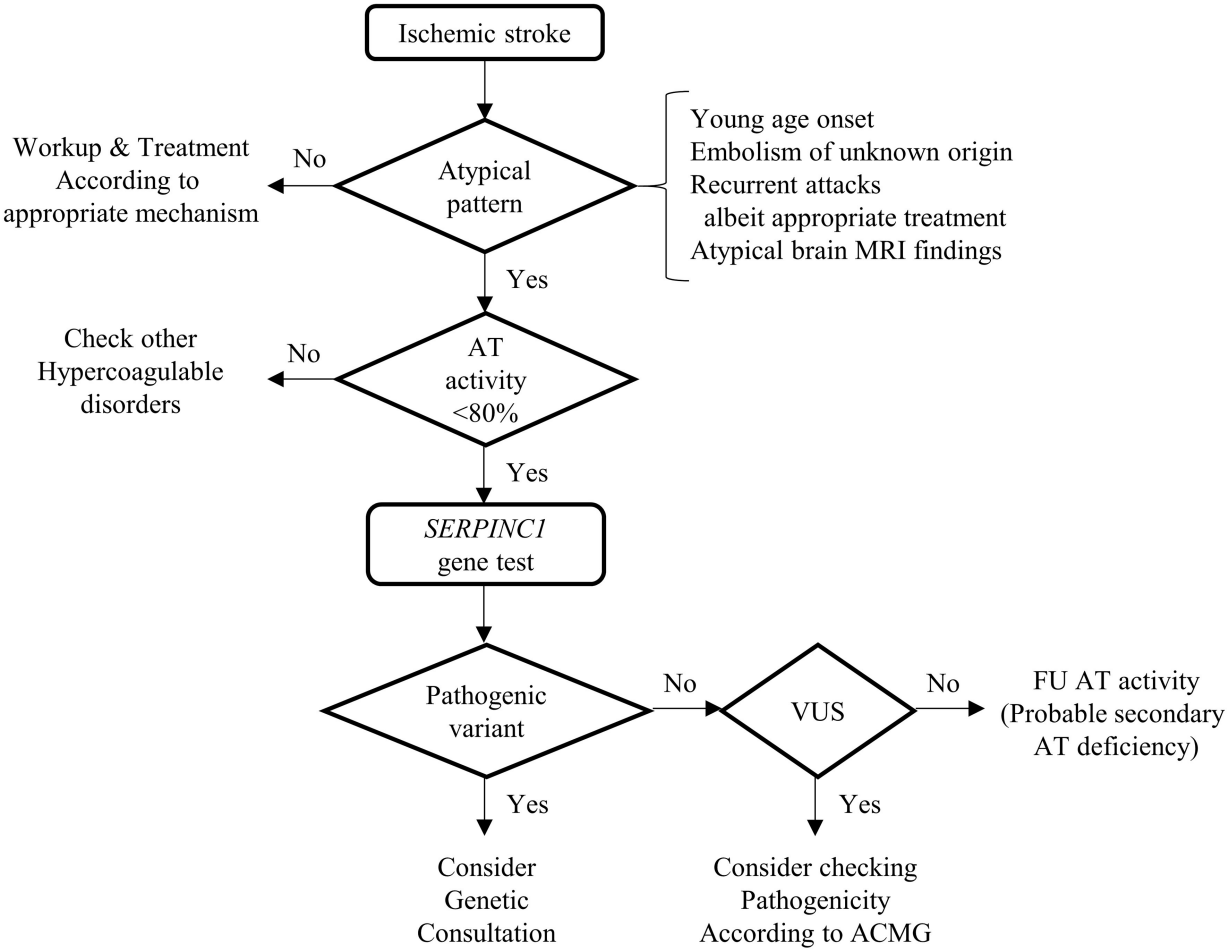
Diagnostic algorithm of *SERPINC1*-associated ischemic stroke. This figure shows the diagnostic process of *SERPINC1*-associated ischemic stroke. The sequential steps will lead to the detection of the pathogenic variants of the *SEPINC1* gene. Because there is the possibility that the variants of uncertain significance could be novel pathogenic, consider checking pathogenicity according to American College of Medical Genetics and Genomics. If the variants are benign, follow up antithrombin activity with enough time interval because the antithrombin deficiency could be secondary, acquired. MRI, Magnetic resonance imaging; AT, antithrombin; VUS, variants of uncertain significance; ACMG, American College of Medical Genetics and Genomics; FU, follow-up.

In the symptomatic AT deficiency with *SERPINC1* gene test cohort, ischemic stroke showed a significant association with the detection of pathogenic variation on *SERPINC1* gene test, which was 100% in the current study. Even though we failed to demonstrate the connection between any kind of arterial thrombosis and the pathogenicity of the *SERPINC1* gene, the detection rate was 87.5%, which was higher than that of the venous thrombosis group (54.5%). Given that various situations could provoke AT deficiency, (2) AT deficiency alone does not ensure the presence of the pathogenic variants of *SERPINC1*. However, the combination of ischemic stroke and AT deficiency was associated with the high detectability of pathogenic variants in the *SERPINC1* gene. In this regard, we propose a diagnostic algorithm for *SERPINC1*-associated ischemic stroke (Figure 1). First, we should check the AT level in the case of atypical ischemic stroke or TIA. The suspicious situations of atypical ischemic stroke include young age, recurrent stroke, or TIA despite appropriate treatment, embolism of an unknown source and atypical findings of brain magnetic resonance imaging (MRI). In particular, when AT activity is low (<80%), the patient should undergo *SERPINC1* gene analysis.

In Korea, the population frequency of *SERPINC1* mutations was reported to be 0.48% (13/3,046) (9). The predominant mutations were *Arg79Cys* and *Ser148Pro*, which are compatible with our findings. In the study, missense mutations of *SERPINC1* were reported. In contrast, our cohort had missense, deletion, and splicing mutations. Although Luxembourg et al. demonstrated that missense mutations tend to develop arterial thrombosis than null mutations, the trend could not be determined in our cohort because of small sample size (4) Nevertheless, the deletion mutations were only found in the venous thrombosis group (Supplementary Tables S1, S2).

Recently, many genetic studies of stroke patients have been reported. In addition to previously well-known monogenic stroke, novel single-gene disorders were discovered (10). Furthermore, genome-wide association studies (GWASs) identified at least 35 loci that significantly increase the risk of stroke (11). Whereas stroke hereditability was estimated to be 30–40%, lead variants explain only 1–2% of stroke hereditability. In all strokes, *SH2B3* was the most significant causal gene. When stratified by stroke mechanisms, *HDAC9* has the strongest association with large artery stroke, and *PITX2* has a close link with cardioembolic stroke. Meanwhile, many variants also influence other vascular traits, such as hypertension, coronary artery disease, moyamoya disease, atrial fibrillation, and venous thromboembolism. Likewise, we suggest that mutation of the *SERPINC1* gene could have close links with stroke, especially atypical stroke mechanisms and vascular traits.

Traditionally, a family history of hypercoagulability is thought to be a predictor of inherited thrombophilia. Nonetheless, the number of patients with a family history of hypercoagulability in our series was too small (2/19, 10.5%). Since *SERPINC1* screening and AT activities were not done in relatives, it was uncertain whether the variants showed variable penetrance or the family members were asymptomatic despite low AT III activity. In some patients, the reason for the paucity of family history could be inferred. The three patients with ischemic stroke (#1, #2, #7) have a variant of p. Arg79Cys. The heterozygote of the variant causes type II heparin-binding site (HBS) deficiency (AT Toyama) and has a mild clinical impact with moderately decreased AT activity (around 50%) (12). However, the three patients showed severe thrombophilia, representing severe AT deficiency (7–27%). Furthermore, two patients had inferior vena cava (IVC) atresia, the manifestation of AT deficiency that has recently been reported in very severe thrombophilia patients with *SERPINC1* p.Leu131Phe, Budapest 3, homozygotes (13). It resulted from the fact that the two patients were homozygous for the Toyama variant, and the other was the compound heterozygote consisting of the Toyama variant and p.Ser148Pro, highly frequent variants with the probable founder effect. Meanwhile, the relatives with p. Arg79Cys heterozygote could be asymptomatic due to mild clinical impact. Otherwise, additional conditions beyond the level of AT might be required to provoke the thrombotic event.

In our cohort, the patients had other risk factors for stroke. Two patients had a cardiac problem. One was an atrial septal defect with conduction abnormalities, and the other was atrial fibrillation. Moreover, anti-phospholipid antibodies were detected in three patients, including one (#1) who was diagnosed with SLE several years after the onset of stroke. We assumed that AT deficiency conferred an additive or triggering effect on the occurrence of ischemic stroke. Another patient (#7) suffered from an acute myocardial infarction (MI) event several months after ischemic stroke. Considering the young age of the patient (4-year-old), the cardiac problem might be associated with AT deficiency due to the pathogenic *SERPINC1* gene mutation (14).

There are some limitations in our study. The number of patients was small. Additionally, because our center is a tertiary hospital, there is a possibility of selection bias. Although the cohorts of previous studies demonstrated that the proportion of stroke was relatively low, our cohort showed a relatively higher ratio of ischemic stroke (6). However, our study only included the symptomatic patients with the *SERPINC1* variant. When the asymptomatic patients with *SERPINC1* variant (*n* = 5), the symptomatic patients whose *SERPINC1* gene was not tested (*n* = 4), and symptomatic patients whose *SERPINC1* gene tested but AT activity was normal or not tested (*n* = 8), are included, the proportion of stroke is 27.8% (10/36). Even though the ischemic stroke incidence was still higher than the previous reports, arterial thrombosis, especially stroke, might be underestimated. Next, the AT activity, but not the antigen level, was used to identify the deficiency. Therefore, AT deficiency types 1 and 2 were not distinguished. However, *SERPINC1* c.1154-14G>A variant is well-demonstrated, inducing type 1 deficiency (15). The missense mutations of amino acid 114 to 156, the region involved in heparin-binding, could lead to type 2 deficiency (16, 17). As mentioned above, a variant of p. Arg79Cys causes type 2 deficiency (12). Moreover, the *SERPINC1* gene test was not performed for four symptomatic patients with AT deficiency, three ischemic stroke patients, and one venous thrombosis patient. Even though selection bias could occur, we assumed that the ischemic stroke patients could also have the pathogenic *SERPINC1* variants. They consisted of two embolic infarctions with unknown origin and a young age stroke with large artery stenosis and demonstrated consistent AT deficiency, suggestive of inherited form. In contrast, the patient with venous thrombosis showed the recovery of AT activity. Lastly, Bravo-Pérez et al. (18) recently suggested the new concept of congenital thrombophilia, transient AT deficiency. The patients of the congenital thrombophilia showed normal AT activity in at least one sample, but the thrombotic events occurred in transient AT deficiency. *SERPINC1* mutation or N-glycosylation defect was confirmed in the patients. Compared to constitutive AT deficiency, transient AT deficiency was associated with arterial thrombosis and less frequent and later thrombotic complications. The concept of transient AT deficiency could be the limitation of our study, proposing the diagnostic flow, in which the *SERPINC1* gene test is warranted after the decreased AT is confirmed. Nonetheless, in the case of ischemic stroke, the symptoms are sudden, and the patients early visit a hospital. If an atypical ischemic stroke is suspected, additional work-up, including AT functional test, will be done immediately. Therefore, transient AT deficiency can be more captured than other thrombotic events.

## Conclusion

This study first showed the utility of the *SERPINC1* gene test in arterial thrombosis, especially ischemic stroke. We propose the diagnostic flow of *SERPINC1*-related ischemic stroke. Moreover, our study could contribute to the development of *SERPINC1* gene test indications. Future prospective or large cohort studies on the *SERPINC1* gene test and ischemic stroke are warranted.

## Data Availability Statement

The original contributions presented in the study are included in the article/Supplementary Material, further inquiries can be directed to the corresponding author/s.

## Ethics Statement

The studies involving human participants were reviewed and approved by The Institutional Review Board (IRB) of Seoul National University, H-2105-031-1216. Written informed consent to participate in this study was provided by the participants' legal guardian/next of kin.

## Author Contributions

SK collected the raw data, analyzed the data, and wrote and revised the manuscript. K-HJ conceptualized and administered the study. W-JL, JM, and K-HJ critically reviewed the manuscript. All authors contributed to the article and approved the submitted version.

## Funding

This work was supported by grants from the National Research Foundation of Korea (NRF) grant funded by the Korean government (MSIT) (2020R1A2C1100337).

## Conflict of Interest

The authors declare that the research was conducted in the absence of any commercial or financial relationships that could be construed as a potential conflict of interest.

## Publisher's Note

All claims expressed in this article are solely those of the authors and do not necessarily represent those of their affiliated organizations, or those of the publisher, the editors and the reviewers. Any product that may be evaluated in this article, or claim that may be made by its manufacturer, is not guaranteed or endorsed by the publisher.
