# Supplementary material for: Utility of the SERPINC1 Gene Test in Ischemic Stroke Patients With Antithrombin Deficiency
Source: Front Neurol. 2022 Jun 3;13:841934. doi: 10.3389/fneur.2022.841934 (PMC9203840; doi:10.3389/fneur.2022.841934)
Supplement: Supplementary file 1 [file Data_Sheet_1.docx]

| **Supplementary Table 1. Distribution of *SERPINC1* variant and the spectrum of phenotype** | | | | | | | | | | | |
| --- | --- | --- | --- | --- | --- | --- | --- | --- | --- | --- | --- |
| ***SERPINC1 gene*** | | |  | **Total (n=13)** |  | **Phenotype** | | | | | |
| **Exon**  **/IVS** | **Nucleotide** | **Protein** | **Type of**  **Mutation** | **Number (Proportion)** | **AT III activity (%)** | **Venous** | **DVT** | **PTE** | **CVT** | **SVT** | **Arterial**  **(=Ischemic stroke)** |
| E2 | c.235C>T | p.Arg79Cys | Missense | 3  (23.1%) † | 19.00  (sd 10.58) | 1  (33.3%) | 1  (33.3%) |  |  |  | 3  (100%) |
| E2 | c.284A>G | p.Tyr95Cys | Missense | 2  (15.4%) | 38.50  (sd 2.12) | 2  (100%) | 2  (100%) | 1  (50%) |  |  |  |
| E2 | c.380G>T | p.Cys127Phe | Missense | 1  (7.7%) | 54.00 | 1  (100%) | 1  (100%) | 1  (100%) |  |  |  |
| E3 | c.442T>C | p.Ser148Pro | Missense | 4  (30.8%) † | 31.75  (sd 16.68) |  |  |  |  |  | 4  (100%) |
| E3 | c.504delA | p.Lys168Asnfs*5 | Deletion | 1  (7.7%) | 40.00 | 1  (100%) | 1  (100%) |  | 1  (100%) |  |  |
| IVS5 | c.1154-14G>A | NA | Splicing | 1  (7.7%) | 60.00 | 1  (100%) |  |  |  | 1  (100%) | 1  (100%) |
| E6 | c.1207del | p.Ala403Hisfs*5 | Deletion | 1  (7.7%) | 44.00 | 1  (100%) |  |  | 1  (100%) |  |  |
| E7 | c.1273C>T | p.Arg425Cys | Missense | 1  (7.7%) | 32.00 | 1  (100%) | 1  (100%) | 1  (100%) |  |  |  |
| † A patient was compound heterozygote consisting of *SERPINC1* c.235C>T and c.422T>C. Two patient was homozygous for *SERPINC1* c.235C>T. Other patients were heterozygote. IVS: intervening sequence; AT: antithrombin; DVT: deep vein thrombosis; PTE: pulmonary thromboembolism; CVT: cerebral venous thrombosis; SVT: splanchnic vein thrombosis; E: Exon; sd: standard deviation | | | | | | | | | | | |

| **Supplementary Table 2. The profiles of pathogenic *SERPINC1* variants with arterial thrombosis versus venous thrombosis** | | | |
| --- | --- | --- | --- |
|  | | Arterial thrombosis (n=7)† | Venous thrombosis (n=8)† |
| Age | | 40.29 (sd 29.81) | 29.75 (sd 14.56) |
| Gender | | M:F = 3:4 | M:F = 5:3 |
| AT activity (%) | | 33.86 (sd 16.84) | 41.75 (sd 10.89) |
| Mutation | |  |  |
|  | Homozygotes | 2 (28.6%) | 1 (12.5%) |
|  | Compound heterozygote | 1 (14.3%) | 0 (0.0%) |
|  | Heterozygote | 4 (57.1%) | 7 (87.5%) |
|  |  |  |  |
|  | Missense | 6 (85.7%) | 6 (75.0%) |
|  | Deletion | 0 (0.0%) | 2 (25.0%) |
|  | Splicing | 1 (14.3%) | 0 (0.0%) |
| IVC anomaly | | 2 (28.6%) | 0 (0.0%) |
| Kidney anomaly | | 2 (28.6%) | 0 (0.0%) |
| APS antibodies | | 3 (42.9%) | 1 (12.5%) |
| Protein C activity (%) | | 99.29 (24.97) | 89 (sd 31.91) |
| Protein S activity (%) | | 82.57 (22.26) | 80.63 (36.46) |
| Family history | | 0 (0%) | 2 (25.0%) |
| †Two patients were included in both arterial and venous thrombosis groups. One was homozygous for c.235C>T, p. Arg79Cys, and the other was c.1154-14G>A, (IVS5), heterozygote (splicing mutation)  sd: standard deviation; M: male; F: female; IVC: inferior vena cava; APS: antiphospholipid syndrome | | | |

| **Supplementary Table 3. The profiles of symptomatic AT deficiency patients with pathogenic *SERPINC1* variant versus without** | | | |
| --- | --- | --- | --- |
|  | | Pathogenic variant (n=13) | Wild type or VUS (n=6)† |
| Age | | 33.46 (23.10) | 37.67 (19.99) |
| Gender | | M:F = 8:5 | M:F = 3:3 |
| AT activity (%) | | 37.23 (13.39)* | 60.00 (19.71)* |
| Venous thrombosis | | 8 (61.5%) | 5 (83.3%) |
|  | DVT | 6 (46.2%) | 3 (50.0%) |
|  | PTE | 3 (23.1%) | 4 (66.7%) |
|  | CVT | 2 (15.4%) | 1 (16.7%) |
|  | SVT | 1 (7.7%) | 0 (0.0%) |
|  | IJC thrombosis | 0 (0.0%) | 1 (16.7%) |
| Arterial thrombosis | | 7 (53.8 %) | 1 (16.7%) |
|  | Ischemic stroke | 7 (53.8%)* | 0 (0.0%)* |
|  | SAT | 1 (7.7%) | 1 (16.7%) |
| Heart problem | | 2 (15.4%) | 0 (0.0%) |
| IVC anomaly | | 2 (15.4%) | 0 (0.0%) |
| Kidney anomaly | | 2 (15.4%) | 0 (0.0%) |
| APS antibodies | | 4 (30.8%) | 1 (16.7%) |
| Family history | | 2 (15.4%) | 0 (0.0%) |
| *p-value <0.05  † The patient with VUS was twenty-years-old female, and the *SERPINC1* gene was a variant of c.1153+5G>T, heterozygote.  AT: antithrombin; VUS: variant of uncertain significance, DVT: deep vein thrombosis; PTE: pulmonary thromboembolism; CVT: cerebral venous thrombosis; SVT: splanchnic vein thrombosis; IJC: internal jugular vein; SAT: splanchnic artery thrombosis; IVC: inferior vena cava; APS: antiphospholipid syndrome | | | |
